# Beyond Plan-Do-Study-Act cycle – staff perceptions on facilitators and barriers to the implementation of telepresence robots in long-term care

**DOI:** 10.1186/s12913-023-09741-9

**Published:** 2023-07-19

**Authors:** Joey Wong, Erika Young, Lillian Hung, Jim Mann, Lynn Jackson

**Affiliations:** UBC IDEA Lab, School of Nursing, University of British Columbia, Vancouver, BC Canada

**Keywords:** Qualitative, Technology, Innovations, Healthcare, Implementation science, Older adults, Virtual communication, Quality improvement, Consolidated framework for implementation research

## Abstract

**Background:**

Quality improvement (QI) programs with technology implementations have been introduced to long-term care (LTC) to improve residents’ quality of life. Plan-Do-Study-Act (PDSA) cycle is commonly adopted in QI projects. There should be an appropriate investment of resources to enhance learning from iterative PDSA cycles. Recently, scholars explored possibilities of implementation science (IS) with QI methods to increase QI projects’ generalisability and make them more widely applicable in other healthcare contexts. To date, scant examples demonstrate the complementary use of the two methods in QI projects involving technology implementation. This qualitative study explores staff and leadership teams’ perspectives on facilitators and barriers of a QI project to implement telepresence robots in LTC guided by the Consolidated Framework for Implementation Research (CFIR).

**Methods:**

We employed purposive and snowballing methods to recruit 22 participants from two LTC in British Columbia, Canada: operational and unit leaders and interdisciplinary staff, including nursing staff, care aides, and allied health practitioners. CFIR was used to guide data collection and analysis. Semi-structured interviews and focus groups were conducted through in-person and virtual meetings. Thematic analysis was employed to generate insights into participants’ perspectives.

**Results:**

Our analysis identified three themes: (a) The essential needs for family-resident connections, (b) Meaningful engagement builds partnership, and (c) Training and timely support gives confidence. Based on the findings and CFIR guidance, we demonstrate how to plan strategies in upcoming PDSA cycles and offer an easy-to-use tool ‘**START**’ to encourage the practical application of evidence-based strategies in technology implementation: **S**hare benefits and failures; **T**ailor planning with staff partners; **A**cknowledge staff concerns; **R**ecruit opinion leaders early; and **T**arget residents’ needs.

**Conclusions:**

Our study offers pragmatic insights into the complementary application of CFIR with PDSA methods in QI projects on implementing technologies in LTC. Healthcare leaders should consider evidence-based strategies in implementing innovations beyond PDSA cycles.

**Supplementary Information:**

The online version contains supplementary material available at 10.1186/s12913-023-09741-9.

## Background

### Implementing technology for quality improvements in long-term care

**Fig. 1:**
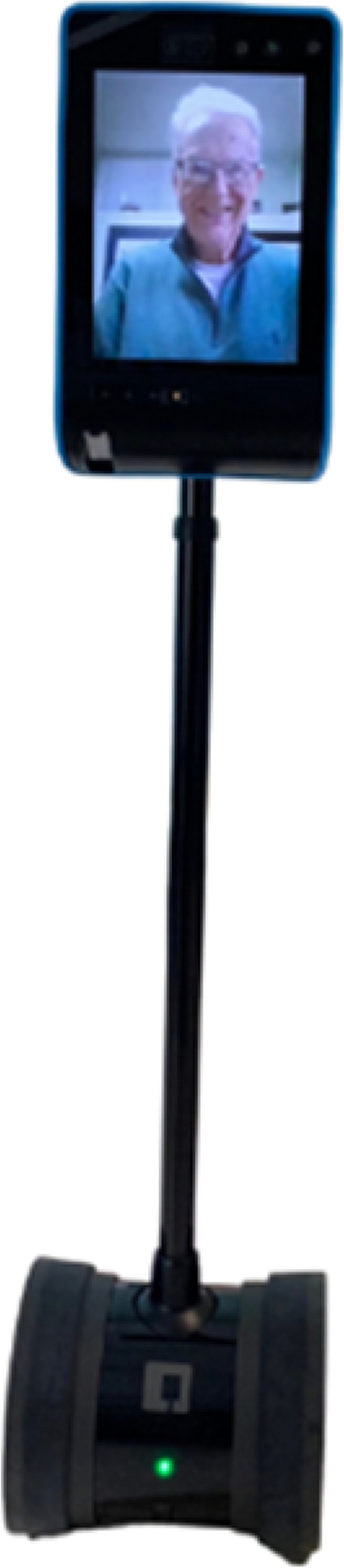
A telepresence robot consists of a tablet and wheel attachments

During COVID-19, residents’ quality of life in long-term care (LTC) has been disproportionately affected. The visitation restriction mandates have increased the likelihood of residents, especially those living with dementia, experiencing social isolation and loneliness [1, 2]. Social isolation correlates with multiple health risks, including depression and cognitive decline [1]. Technology for virtual family visits, e.g., telepresence robots, has been introduced in LTC to address social isolation. Telepresence robots are tablets for video conferencing, allowing the end user to remotely control movement with wheel attachments (see Fig. 1 for an example of a telepresence robot). Using these robots has been one of the ways researchers have explored to facilitate social connections in care settings [3]. Some benefits of telepresence robots were their ease of use, mobility functions, and feeling of the presence of remote individuals [4, 5]. There is a need for frontline and operational leaders to explore innovative strategies to implement useful technologies to continuously improve the quality of care and experiences of residents and family members in LTC.

To successfully implement and integrate technological innovation in a complex healthcare environment, factors of multiple levels (e.g., patients, organizational, and policy levels) need to be systematically considered to smoothen the implementation processes [6]. However, there are factors of implementation that have not been fully explored, e.g., the perspectives of frontline staff and leaders [7]. Dyb et al. [8] suggested a more in-depth exploration of staff’s visions would facilitate technology planning and implementation. Franke et al. [9] stated the importance of exploring the role of leadership in technology implementation. Having frontline staff to facilitate quality improvement with a lack of training and support from the management level was associated with the failure of the change process [7]. A recent scoping review by Hung et al. [3] identified that no implementation frameworks were used in planning or evaluating the implementation of telepresence robots in care settings. The stakeholders’ perspectives on robot adoption have not been fully explored. Investigations on strategies to support effective quality improvement programs, e.g., implementing technologies in healthcare settings, are needed.

### The Plan-Do-Study-Act cycle in the healthcare context

One commonly adopted quality improvement (QI) method in healthcare settings is the Plan-Do-Study-Act (PDSA) cycle. It is a four-stage cycle that originated from industrial settings [7]. PDSA changes are often small and focused on a microsystem and a unit setting. The processes allow learning and explorations into how the microsystem reacts to the implemented intervention [10]. In healthcare, PDSA cycles support iterative processes for making changes and improvements [11]. The first stage of the cycle, ‘Plan’, is to identify an objective or form a hypothesis for improvements. ‘Do’ is the stage to execute the plan, collect data, and document problems and observations. During the ‘Study’ stage, the QI team will analyze the data collected in the previous stage by comparing it to the predictions made earlier and summarize what is learnt from the results. The last stage is ‘Act’, the team will decide what changes to be made in the next cycle or not to proceed with more interactive cycles [11]. The QI team will complete multiple cycles as required [7]. The team can learn from the processes of the completed PDSA cycles, no matter whether the QI projects are successful or not in the settings.

### Investment of resources before the start and within PDSA cycles

Although the PDSA method provides a basic four-step approach with a nature of simplicity, a proper investment of resources is essential at each stage of the cycle. Having successful and sustainable quality improvements using the PDSA cycle has been challenging within complex healthcare settings due to the underinvestment of time and resources in the stages of the cycle [7]. Before the start of a cycle, exploration needs to be done to engage stakeholders to identify the problems and possible interventions correctly. Without early stakeholders’ engagement, there will be predictably less shared ownership of successful change by staff and easy disengagement [7, 12]. At the ‘Plan’ stage, there is often limited time spent on planning the implementation, data collection and analysis processes on who, where and when to implement the intervention. Moreover, there were many instances where ‘Do’ was prioritized, diminishing the importance of putting resources into ‘planning’, and ‘studying’ [7]. Furthermore, after analyzing what has been learnt from the ‘Study’ stage, miscommunication or failures to communicate study results will lead to disruptions in the subsequent cycles or projects. For ‘Act’, sometimes the team moves too quickly from small-scale tests to full-scale implementation, which may lead to project failures and de-implementations [7]. Investing appropriate time prior to and within the cycles will avoid wasted effort by the team and facilitate achieving QI goals.

### The collaborative approach of quality improvement (QI) and implementation science (IS)

Besides addressing the inappropriate applications of PDSA cycles with more human resources and financial support to reach QI goals [7], scholars [10, 13, 14] have explored the possibilities of applying implementation science (IS) with QI methods to enhance the generalisability of QI projects to other contexts in healthcare services.

IS is based on behavioural changes and focuses on making findings generalizable [10]. The Consolidated Framework for Implementation Research (CFIR) is a conceptual framework in IS developed based on multiple theories that guide implementors to systematically map various contextual factors that may impact the intervention implementation [6]. The Framework consists of five domains (Intervention Characteristics, Outer Setting, Inner Setting, Characteristics of Individuals, and Process) (see Table 1 for the descriptions of the CFIR domains) and 37 constructs [6, 15, 16]. CFIR is a practical guide for implementors to systematically assess multi-level potential barriers and facilitators for interventions’ implementation (planning, executing, and evaluating) [17]. Recent research by Smith-Miller [18] demonstrated how to use CFIR to analyze factors from nurses’ perspectives that influenced the implementation of evidence-based guidelines in clinical settings. Furthermore, Milton et al. [19] used CFIR to evaluate the implementation strategies of a colorectal cancer risk prediction tool in primary care.

**Table 1:** Descriptions of five CFIR domains

Name of domains	Description
Intervention characteristics (IC)	Includes constructs that describe features of the interventions that impact the success of implementation, e.g., adaptability and complexity [20]
Inner setting (IS)	Includes constructs that describe characteristics of the organization where the innovation is implemented, e.g., compatibility and leadership engagement [21]
Outer setting (OS)	Includes constructs that describe the external impact on the implementation of an intervention, e.g., patient needs and resources [21]
Characteristics of individuals (COI)	Includes constructs that describe actions and behaviours of individuals in the organization that interplay with the setting and the organization, e.g., knowledge and beliefs about the intervention and self-efficacy [22]
Process (P)	Includes constructs that describe implementation stages and key stakeholders, e.g., planning, reflecting and evaluating, opinion leaders [21]

IS can contribute to the development of interventions, and planning and evaluation of implementation in QI projects to make the work more easily spread to other contexts [10]. The strategies- and framework-driven designs of IS allow systematic assessment and reporting of adaptations of interventions [10]. There have been some examples of the pairing of CFIR with PDSA cycles in QI projects [14, 23]. CFIR was applied to successfully guide the planning, design, and evaluation prior to and within a PDSA cycle in a quality improvement initiative to increase pain management referrals for youth with sickle cell disease. The results helped the team carry out subsequent PDSA cycles [14]. In another example, CFIR was used after two PDSA cycles to systematically analyze facilitators and barriers to a QI program to reduce missed opportunities for vaccination in five primary healthcare facilities. The feedback and information generated were valuable to informing and adjusting the subsequent cycles to achieve QI goals [23].

### Our study as an example of pairing CFIR and PDSA cycle in technology implementation in long-term care

Although the research mentioned above [14, 23] showed positive contributions of using CFIR and PDSA methods collaboratively on facilitating effective PDSA cycles in QI programs, there have been few examples to demonstrate the pairing of IS and QI methods in healthcare settings, particularly in the technology implementation.

The purpose of this article was to systematically investigate the context of a QI program – implementing telepresence robots to improve residents’ quality of life in LTC. The CFIR-guided exploration of the perspectives of multidisciplinary staff and leadership teams helped identify perceived facilitators and barriers to adopting telepresence robots in LTC and plan possible strategies for different stages in the upcoming PDSA cycles. The study can contribute further insights into how CFIR can complement PDSA cycles in QI programs involving technology implementation in healthcare settings.

## Methods

### Design

We employed a qualitative approach in data collection. Qualitative research offers insights into how an intervention works or does not work and what facilitates and hinders its implementation in a complex healthcare context [20]. This qualitative descriptive study [24] enables us to explore multidisciplinary frontline staff and leadership teams’ perspectives on facilitators and barriers to implementing telepresence robots in LTC. The Standards for Reporting Qualitative Research (SRQR) [25] (See Additional file 1) was used to guide the reporting of qualitative findings.

Our team consists of five members: two people living with dementia, one researcher in nursing, and two graduate students. We valued patient engagement in research and involved patient partners with lived experiences in different stages of the research process. We worked as a team in planning the interview guide, co-facilitating interviews and focus groups, data analysis and co-authoring the manuscript.

Research Questions:

1. What are the anticipated facilitators and barriers to implementing telepresence robots in LTC from the perspectives of frontline staff and leaders in LTC in Canada?
2. What are the possible strategies that help overcome the barriers to adopting the robots for virtual family visits?

### Setting

Participants were recruited from two LTC in British Columbia, Canada. Both LTC are publicly funded and occupied by residents with complex care needs. Most residents (over 80%) are older adults with cognitive impairment or dementia. Both sites offer single-occupancy rooms for their residents.

### Recruitment

We used purposive and snowball sampling methods to recruit participants. The staff of different disciplines and operational leaders were recruited with the assistance of local educators. The local educators sent emails and posted posters to invite staff to participate in the research. The emails and posters provided the study information and inclusion criteria. A total of 22 participants were recruited. Participants included operational and unit leaders and interdisciplinary staff, including nursing staff, care aides, and allied health practitioners. After 22 participants were interviewed, we gained sufficient information to answer the study questions. See Table 2 for the characteristics of staff participants.

### Inclusion/ exclusion criteria

The inclusion criteria for participants were: (a) regular or casual staff of any disciplines, administrative staff, or operational and unit leaders, and (b) currently working in either of the two LTC.

**Table 2:** Demographic characteristics (*n* = 22)

Characteristic	n (%)
**Sex**	
Male	8 (36.4)
Female	14 (63.6)
**Current role in LTC**	
Operational leader	9 (40.9)
Patient care coordinator	1 (4.6)
Registered nurse	1 (4.6)
Care aide	4 (18.2)
Occupational therapist	1 (4.6)
Physiotherapist	1 (4.6)
Social worker	1 (4.6)
Rehabilitation and activity assistant	4 (18.2)

### Data collection

#### Using CFIR to develop the interview guide

Based on the research questions, our team developed the interview guide together. We reviewed the CFIR Interview Guide Tool [17] together to select relevant domains and constructs. Not all constructs were chosen as some were irrelevant (e.g., Cosmopolitanism in the Outer Setting domain which explores the organization network with other external organizations). As a team, we selected relevant guiding questions within the chosen constructs. Then, we developed the four guiding questions and follow-up questions for each guiding question (see Table 3 for the development of the interview guide).

**Table 3:** Development of the interview guide

Research questions	Select relevant constructs and suggested questions from the CFIR Interview Guide Tool *(Using (1) Intervention Characteristics (Relative Advantages) and (2) Outer Setting (Patient Needs & Resources) as examples)*		Generate interview questions in the context for telepresence robot implementation	Follow-up questions based on the suggested relevant questions
1. What are the anticipated facilitators and barriers to implementing telepresence robots in LTC from the perspective of frontline staff and leaders in LTC in Canada? 2. What are the possible strategies that help overcome the barriers to adopting the robots for virtual family visits?	**Domain**: Intervention Characteristics (IC) **Construct chosen**: Relative Advantages	**Questions selected**: 1. How does the intervention compare to other similar existing programs in your setting? 2. How does the intervention compare to other alternatives that may have been considered or that you know about?	1. What are the usual ways for resident-family communication and staff-family communication at your workplace?	1.1 How are telepresence robots different when compared to these usual ways of communications? (IC - Relative advantages) What are the challenges of using these existing ways of communication? 1.2 To what extent are new ideas adopted and used to make improvements at your workplace? (IS – Culture)
	**Domain**: Outer Setting (OS) **Construct Chosen**: Patient Needs & Resources	**Questions selected**: 1. How well do you think the intervention will meet the needs of the individuals served by your organization? 2. How do you think the individuals served by your organization will respond to the intervention? 3. What barriers will the individuals served by your organization face to participating in the intervention?	2. How can telepresence robots help residents with dementia?	2.1 What are these residents’ needs? And how do the robots meet the needs of residents? (OS – Patient Needs & Resources) 2.2 How do you feel when you picture yourself or residents using the robots? (COI - Knowledge & beliefs about the intervention) 2.3 How do you feel the residents will respond to the telepresence robot? (OS – Patient Needs & Resources)
			3. What difficulties (may) happen when using telepresence robots at your workplace/ in a long-term care setting?	3.1 What difficulties will the residents face in using telepresence robots? (OS – Patient Needs & Resources) 3.2 How well does the telepresence robot work with existing working processes and routines in your setting? (IS - Adaptability) 3.3 What support would you prefer to help you implement and use the robot? (IS – Readiness for Implementation) 3.4 How will the infrastructure (physical layout, technical support) affect the use of telepresence robots? (IS – Structural characteristics) 3.5 How well does the telepresence robot fit with existing work processes and routines in your setting? (IS – Implementation climate) 3.6 How confident are you that you will be able to use the Telerobot? (COI – Self-efficacy) **Extra questions for leadership teams**: 3.7 What (cost) will be considered when deciding to implement an intervention? (IC – Cost) 3.8 Do you expect to have sufficient resources to use the Telerobot? (IS – Readiness for Implementation)
			4. What will lead your workplace/ LTC setting to keep the telepresence robots in use?	4.1 Can you describe a recent implementation of a new program? (IS - Learning climate) 4.2 Who are the key individuals to get on board with the use of telepresence robots? (P - Engaging) 4.3 What policies, restrictions or guidelines would affect the use of the robot? (IS – Readiness for implementation) 4.4 What kind of support or actions can you expect from leaders to help to use Telerobot at your workplace? (IS – Readiness for implementation) 4.5 What steps are usually taken to encourage staff to commit to using a new technology/ to a change in your organization? (P – Planning) **Extra questions for leadership teams**: 4.6 How well does the telepresence robot fit the values and norms in your setting? (IS – Implementation climate)

#### Interviews and focus groups

We conducted both interviews and focus groups via Zoom virtual or in-person meetings to accommodate the staff workflow in clinical settings and based on their preferences. Five interviews and eight focus groups, which lasted for 20–30 min each, were digitally recorded and conducted between November 2021 to December 2021 via Zoom virtual or face-to-face meetings. All in-person interviews and focus groups were co-facilitated by the first and second authors in the unit’s dining area. The first author paired up with a team member living with dementia (the fourth and fifth authors) to co-facilitate the virtual interviews and focus groups. The interviews and focus groups were semi-structured. At the beginning of the interviews, all interviewees watched a one-minute video clip of the interactions between a resident with the telepresence robot to give them a brief understanding of how the robot worked in the healthcare context. Participants were asked about the usual resident-family communication methods at their care homes as baseline information. For the focus groups and interviews co-facilitated by the first and the second authors, the first author asked the four guiding questions, while the second author asked follow-up questions for each guiding question. The second author was also the note-taker. For the virtual interviews by the first author and the team member living with dementia, the team member asked the guiding questions and the first author asked the follow-up questions. The first author was the note-taker for these interviews. In all interviews, the first author was responsible to play the video clip. After each interview or focus group, the co-facilitators would have a 15-minute debriefing session to discuss significant findings, observations and to prepare for the next interviews.

#### Research ethics

Institutional ethics approval was obtained from the principal investigator’s University Research Ethics Board (H21-00844). Participants were given written information about the study, and all participants signed consent before the interviews. Participation was voluntary. All participants received a $20 gift card as an incentive. We have used pseudonyms throughout the reporting of data to maintain confidentiality.

### Data analysis

All recordings were transcribed verbatim and checked by the first and second authors. The first author reviewed the data, used thematic analysis [26] to generate initial codes, and grouped them manually without using analytic software. Inductive codes were developed based on empirical data, and deductive codes were drawn from constructs in CFIR [17]. The first three authors reviewed, refined, and agreed on a general coding framework. Then, the first author developed categories and initial themes. The entire team was involved in the subsequent refining cycles to ensure rigour. With the diverse perspective of multidisciplinary healthcare professionals and patient partners, we critically reflected, discussed, questioned, and refined the categories and themes. We related the categories with CFIR domains and constructs (See Table 4 for the development of themes based on categories and codes of original quotation examples). All researchers agreed on the final structure of themes and quotes included. The whole team then discussed how the results could support planning the PDSA cycle.

**Table 4:** Development of themes

Example of original text/ quotations	Codes	Categories	Themes	Related CFIR domain and construct
“The robots are now more urgently needed when in-person visits are not allowed.”	Residents’ needs; Perceived advantages; Tension for change; Facilitator	Restrictions in LTC increase the need for telepresence robots.	‘The robots are now more urgently needed.’- the essential needs for family-resident connections	IS – Tension for Change OS – Patient Needs & Resources COI – Knowledge and Beliefs about the intervention
“To listen to the voice of the frontline staff…because it does not matter if leaders think the device is good.”	Staff attitude; Organizational decision priority; Staff engagement; Facilitator; Barrier	Staff perceptions impact leadership commitment.	‘Listen to the voice of frontline staff’- meaningful engagement builds partnership	IS – Leadership Engagement IS – Relative Priority COI – Knowledge and Beliefs about the intervention
“I am a visual learner. I need to practice and practice a few more times before I get familiarized with how to do it. Practice is critical besides verbal or written education.”	Staff attitude; Self-efficacy; Learning styles; Increased familiarity; Facilitator	Practice opportunities increase confidence.	‘I need to practice and practice’ – training and timely support gives confidence	COI – Self-efficacy COI – Other Personal Attributes P – Planning

## Results

Twenty-two participants expressed their views about implementing telepresence robots in LTC. Most participants were female (63.6%). Over half (59.1%) of the participants were frontline staff of various disciplines (e.g., registered nurses, care aides, and rehabilitation assistants). The remaining participants were operational leaders (40.9%). All participants did not have experiences of using telepresence robots before and were not familiar with the use of telepresence robots in healthcare context.

The analysis revealed three themes: (a) *‘The robots are now more urgently needed.’* - the essential needs for family-resident connections, (b) *‘Listen to the voice of frontline staff’*- meaningful engagement builds partnership, and (c) *‘I need to practice and practice’* - training and timely support gives confidence.

### Theme 1: ‘The robots are now more urgently needed.’- the essential needs for family-resident connections

The most common positive impacts of telepresence robots in LTC anticipated by participants were socialization and social connection. Many staff expressed the perceived for telepresence robots to facilitate social connection for residents during the periods of lockdown and visitation restriction policies in LTC during the pandemic. Katherine (female) said, *“The robots are now more urgently needed when in-person visits are not allowed.”* Staff also shared examples of family members who could not visit the residents in LTC due to their unvaccinated status. The limitations of in-person visits create tensions for change to adopt these robots in care homes [27]. Staff also recognized that telepresence robots could help address geographically challenging situations for family members. Janet (female) stated, *“Some family members are living abroad. Telepresence robots open so much in terms of connections. The robots address these families’ anxiety and concerns as it is a hard decision for family members to place somebody in a care home.”*

The extent of patient needs and how the innovation meets those needs can potentially facilitate or impede implementation [OS – Patient needs and resources] [6, 28]. Amy (female) suggested the facilitation by telepresence robots for interpretation, *“For a person who speaks a different language, staying connected with somebody who understands them [through the robot] would be really good.”* Staff stated the unique advantages of telepresence robots for social connections when compared to other devices, which often required staff technical assistance during use. Lily (female) said, *“The robot turns on and off itself [as call ends] …no need to be ‘set up’… Residents don’t need to ‘work’ the robot like they have to ‘work’ the tablet, which many do not know how to… and staff don’t have to help residents use the robot like how they would have to help with the tablet.”* Mandy (female) commented on how robots could potentially support private resident-family conversations, *“Robots can create private spaces for family and residents for their conversations. They can share intimate information without staff assisting and staying in the room.”* Having relative advantages recognized and acknowledged by key stakeholders are effective in implementation [IC – Relative advantages] [29]. However, some staff added that telepresence robots might not suit all residents. *“If we need to assist and give cues to those residents who are not good at interacting in the conversation, it will take us much time and create burdens. We still need to care about other residents,”* Rocky (male) expressed.

Besides relative advantages, the more the individuals’ value aligns with the perceived value of interventions, the more successful the implementation will be [IS – Compatibility] [30]. Some participants shared how the anticipated benefits of family-resident connections aligned with their values, which overcame the concerns about increased workload. Katherine (female) expressed, *“We need to be flexible; we cannot refuse to help because those tasks are not listed in our job duties. We want to help residents as they can connect with their family members to address their emotional needs.”* Johnny (male) added, *“It goes to our philosophy of care, like the belief of involving families in the care of residents… Staff who intrinsically support this philosophy will probably be the adopter.”* One participant expressed that it would be an add-on task for staff who did not value family-resident connections to learn how to use the robots.

Leadership teams also shared how telepresence robots’ perceived needs and benefits were connected to the organization’s values. Alex (male) said, *“This is not about us; this is about residents and families that we support…Finding more ways to support residents to make connection easier is our job to do.”* They considered implementing this technology as it can potentially promote person-centred care by increasing family-resident connections. Janet (female) suggested, *“It’s an opportunity to shift culture and mindset. The foundation is that care homes are not staff’s workplace, but where people live and people’s homes.”* This perceived fit with an organizational mission is a predictor of successful implementation [IS – Compatibility] [30] and engages leaders to support the intervention as it promotes their organizational goals [IS – Leadership engagement] [31].

### Theme 2: ‘Listen to the voice of frontline staff’- meaningful engagement builds partnership

Operational leadership participants expressed that staff buy-in is essential when they consider adopting an intervention in an organization. They emphasized that staff support could outweigh the costs and change leaders’ attitudes toward the interventions. Cindy (female) expressed, *“Staff buy-ins is one factor that drives purchasing decisions of the organization… If frontline staff are asking for the device, and if the device is crucial to the operation of the unit.”* Fred (male) added, *“To listen to the voice of the frontline staff…because it does not matter if leaders think the device is good.”* The implementation can be strengthened if the leadership team shows commitment and active interest [IS – Leadership engagement] [31].

Mandy (female) shared on strategies to meaningfully engage staff, *“Create open spaces to ask about staff concerns and validate them… the closer you are physically to staff, the more engaged and grounded approaches that you use, the more responses that you will get.”* A positive and psychologically safe space and adequate time for reflective thinking and debriefing contribute to shared learning of implementation progresses and experiences [P – Reflecting and evaluating] [32]. Cindy (female) stressed the need to take into account the staff’s perspectives, acknowledging that they might be experiencing “consultation fatigue” due to being consulted on multiple matters. The leader recognized that barriers may exist in engaging staff in planning and implementing, as well as obtaining feedback, especially when there are simultaneous new initiatives requiring staff participation.

In the interviews, staff participants shared concerns about using telepresence robots in the workplace. The common concern was around the intrusion of residents’ privacy. Sophie (female) suggested, *“Certain boundaries and guidelines will be needed to prevent invasion of privacy.”* The other common concern was resource allocation when limited robots were available in LTC settings. Karen (female) explained, *“If there is a limited number of telepresence robots, family members will need to take turns to use them.”* Johnny (male) suggested on training resources, *“More resources will motivate various users to learn how to drive the robot and to plan the robots to be part of their work.”* The level of provision of telepresence robots and the suggested resources of guidelines and training materials to staff can impact the implementation [IS – Available resources] [33].

Participants anticipated that some staff would be reluctant and afraid to try new technologies. Engaging unsupportive staff can negatively impact the likelihood of successful implementation, while support from opinion leaders facilitates the implementation [P – Engaging] [34]. Johnny (male) expressed, “*Engaging those who are interested and see the potential benefits of using the robots*… *Sometimes you just cannot get the buy-ins from certain staff.”* Janet (female) suggested to gain support from multiple sources, *“Get support from different levels of leadership and leaders representing different groups, for example, leaders of the family council, residents’ council, and operational leaders. They can be helpful to generate engagement.”*

### Theme 3: ‘I need to practice and practice’ – training and timely support gives confidence

Many participants emphasized the need for training in implementing telepresence robots in healthcare settings. Easy access to consumable knowledge about the intervention is key to successful implementation [IS – Access to knowledge and information] [35]. Furthermore, many of them preferred hands-on and in-person training over paper manuals. Sophie (female) shared on her learning experiences, *“I am a visual learner. I need to practice and practice a few more times before I get familiarized with how to do it. Practice is critical besides verbal or written education.”* With more exposure to using the robots, participants believed that staff would be motivated to incorporate robots into their workflows. Brian (male) stated, *“If the chance of learning how to use the robot is scant, it is natural for people not to incorporate telepresence robots into their workflow. You will not plan to use it.”* The availability of tailored training about the telepresence robots facilitates building a positive attitude from stakeholders to be committed to the intervention. Lily (female) shared, *“Once we know how to use it, of course, we will keep the robot in use! We need to try it first.”* Vera (female) added, *“If we enjoy using the technology, we will encourage other staff to use it.”*

Besides training, participants appreciated timely technical support for staff. With available technical support, participants expected an increased self-efficacy in using robots. Britney (female) expressed, *“If there are technical issues, staff can contact someone. It will increase the feeling of confidence of staff to use it.”* Confidence in their abilities is associated with a higher sense of self-efficacy, raising acceptance rates of the intervention [COI – Self-efficacy] [36]. Participants also shared the different levels of staff readiness and their perceived intervention complexity. Veronica (female) said, *“Some colleagues told me that it is like playing games on a computer, but I have never played those games. It seems complicated to me.”* The implementation is more likely to be effective if the stakeholders perceive the intervention as straightforward [IC – Complexity] [37].

## Discussion

Using CFIR as an evidence-based guide, our study explores and illustrates anticipated enabling and impeding factors to implementing telepresence robots in LTC based on various stakeholders’ perspectives. The following demonstrates how we can develop possible strategies to overcome barriers and build facilitators for planning different stages in upcoming PDSA cycles after these factors are identified with the help of CFIR. Table 5 summarizes with relevant CFIR domains and constructs and how the evaluation by CFIR can help systematically inform the PDSA plan. An easy-to-use tool, ‘START’ (see Table 6 for the tool), has been developed based on the study’s findings and CFIR. This tool summarizes five possible areas for frontline leaders to consider and invest resources and time in when they plan, implement, and evaluate technological interventions in LTC before, during and after each PDSA cycle.

**Table 5:** Summary of findings based on CFIR domains and constructs

Theme	Summary of data extracted	Facilitator / Barrier	Domain	Construct	Possible strategies to help with planning the PDSA cycle?
1	The robot could support: • Residents’ needs during the pandemic • Families’ geographical needs	Facilitator	Outer Setting	Patient needs and resources	Plan – stress these perceived residents’ needs to increase staff buy-in
	The robot did not match the residents’ readiness to use the intervention	Barrier			Plan – access residents’ needs and whether the intervention match or possibilities to adapt to these needs Study – evaluate whether the intervention is helpful for residents Act – whether the intervention can be adapted
	The robot allowed private conversations compared to other devices	Facilitator	Intervention characteristics	Relative advantage	Plan – stress these relative advantages
	The use of robots aligned with: • Staff personal philosophy of care • Organizational mission	Facilitator	Inner setting	Compatibility	Plan – evaluate staff and organizational values; emphasize these values when introducing the technology; engage those staff who have similar values; promotes the benefits to leadership teams and gain their support
	The use of robots did not align with the staff’s personal values	Barrier			
	The use of robots could promote own organization’s goals	Facilitator		Leadership engagement	
2	Staff buy-in drove leaders/ organizational purchasing decision	Facilitator	Inner setting	Leadership engagement	Do – collect staff successful stories/ positive comments Study – communicate positive comments to leadership teams Act – tailor planning with staff to promote ownership of the project
	There were guidelines to prevent privacy invasion	Facilitator		Available resources	Plan – prepare the guidelines requested by the staff Study – evaluate the usefulness of the guidelines and whether improvements needed
	There was a limited number of telepresence robots for use	Barrier			Plan – co-create with staff the strategies of the sharing use of robots in the settings, e.g., scheduling of calls Do – observe and documents failures or successes of the strategies Study – analyze the data Act – adjust strategies in the subsequent cycle
	There was a dedicated time to debrief and reflect	Facilitator	Process	Reflecting and evaluating	Plan – plan constant check-ins with staff during the cycle to listen to their concerns; build relationships with staff at the beginning and let them know that there is a safe space to talk Do – dedicate time and listen to staff concerns and feedback Study – analyze feedback and assess the acceptability of the robots Act – co-create improvement plans with staff for the next PDSA cycle
	There was an open space to share concerns	Facilitator		Reflecting and evaluating	
	During the implementation process, there was a successful engagement of: • Supportive staff • Implementation leaders from different groups	Facilitator		Engaging	Plan – co-create engagement plans with staff champion and leadership teams who are familiar with the working cultures; learn about who the opinion leaders are in different groups in the LTC; start with engaging the supportive staff Do – engage stakeholders early; observe engagement processes to document which strategies work and which do not Study – evaluate the engagement plan with data collected; communicate what has been learnt
	Unsupportive individuals did not have buy-in for the intervention	Barrier			
3	There was easily accessible training	Facilitator	Inner setting	Access to knowledge and information	Plan – learn about staff’s preferred training style and time; prepare preferred training materials; offer more training sessions if needed Do – documents comments on training materials and training processes Study – analyze the effectiveness of training materials and procedures Act – adjust training sessions and materials
	• There was incompatible training time and length • There were limited chances to try using the robots	Barrier			
	Staff perceived that the intervention was complex	Barrier	Intervention characteristics	Complexity	Plan – provide training; identify staff who need extra help Do – document and acknowledge staff concerns Study – find out what works for them and what do not Act – focus on training strategies that work for them in the subsequent cycles
	Staff had an increased level of confidence in using the robots	Facilitator	Characteristics of individuals	Self-efficacy	

### Plan

Our findings show that spending time and resources investigating residents’ needs, staff, and organizational values facilitates implementation in different CFIR domains. Having perceived residents’ needs and tension for change to adopt the intervention promotes an implementation climate. Understanding residents’ needs and demonstrating how the technology matches residents’ needs can help with gaining staff buy-in [27, 38]. Approach staff who have similar values at the initial stage to secure support. Acceptance of the technologies and motivation to use the intervention will be higher if the aim of using the technologies aligns with the values of individuals and the mission of the organizations [38]. At the planning stage, frontline leaders need to promote the benefits of the interventions that align with the workplace or residents’ needs and staff values. The perceived benefits of addressing residents’ needs will become an internal drive and motivation for staff to adopt using the robots in their routines [39].

Appropriate plans on guidelines and training for using the robots, staff engagement and training, and debriefing sessions are essential. Co-creating guidelines and protocols for implementing the technologies smoothen the implementation process, e.g., sharing the use of robots and scheduling robot calls. Engagement plans can be co-designed with staff champions and leadership teams to fit the working culture and target opinion leaders and supportive staff. Engagement plans can also include promoting the technology in various groups, such as family and resident councils in LTC. Getson and Nejat [40] suggested that all stakeholders should be considered in technology implementation. Identifying champions among staff members was an enabling factor for the successful implementation of technology in LTC homes. Furthermore, it is crucial to plan suitable training. In the interviews, participants suggested some considerations for training designs. Frontline leaders can co-design training with relevant stakeholders and tailor it to individuals’ needs, learning styles, and preferred modes of training [41, 42]. Tailoring training to meet individuals’ needs echoed a study by Yuan et al. [39] that emphasized the diversity of care staff skills and motivations when introducing social robots to staff in LTC.

### Do

Time and resources need to be dedicated to data collection during the intervention implementation process through observations and asking for staff comments. The implementation team needs to observe and document failures and successes, such as which engagement strategies work and which do not. Early engagement of stakeholders in implementation will more likely lead to success and should be valued [34, 43]. They should be involved from the initiation phase of the implementation process. As mentioned by the participants, engaging those who have buy-in and those who have influence with diverse groups is crucial. Frontline leaders need to be mindful of choosing whom to engage at the start [43].

Operational and frontline leaders should dedicate time and provide an open space for the staff to reflect on the implementation process [44] and listen to their concerns and feedback on a diverse aspect of the intervention, e.g., acceptability of the robots and training processes to inform the analysis in the ‘study’ stage. Koh et al. [45] demonstrated how joint discussions with colleagues allowed staff to express their concerns and empower each other with skills to manage the negative reactions of residents using the pet robots.

### Study

Individuals adopting PDSA cycles sometimes fail to evaluate the implementation process [7]. Leaders can use CFIR as a framework to guide them to perform systematic analysis by exploring multidimensional factors, for example, the effectiveness of training procedures, staff engagement strategies and the impact of robots on residents. Sharing successful residents’ stories and having team debriefings of experiences promote shared learning and improvements [32, 44]. From the interviews, we learned that once staff was empowered and enjoyed using the technology, they would share the stories and encourage the use of the technology in their workplace. Communicating positive comments to the leadership teams may also help facilitate leadership support. A study demonstrated the positive changes in nursing staff’s perceptions towards a robotic seal and started incorporating the robot into their routines after observing the therapeutic effects of the robot with the residents in LTC [46].

### Act

Based on the data analysis from the previous stage, a decision will be made on whether to adjust the implementation plans for the next cycle or abandon the intervention and not proceed with the subsequent cycles. Co-creating improvement plans and co-planning the next PDSA cycle recognizes staff involvement and ownership of the QI project, which potentially increases staff buy-in and leadership support to the intervention implementation, e.g., modified training procedures and debriefing sessions. Rubeis [47] also shared how health professionals like nurses could play a more significant role in policymaking and contribute their expertise in how technologies could be implemented in the gerontological field.

### Implication

Based on the findings, analysis, and CFIR, we propose an easy-to-use tool – ‘**START**’ (see Table 6 for the tool) for healthcare leaders to consider before and within the PDSA cycle to promote starting various technological implementations in LTC.

**Table 6:** A tool ‘START’ for the technology implementation in LTC based on empirical findings and literature

	Data extract example	Literature example
**S - Share benefits and failures** Share benefits of interventions that align with the values of staff, have team reflections on successful stories and experiences of residents and staff, and communicate failures to avoid them from happening in the subsequent cycle	- “The robot turns on and off itself [as call ends] …no need to be ‘set up’… Residents don’t need to ‘work’ the robot like they have to ‘work’ the tablet, which many do not know how to… and staff don’t have to help residents use the robot like how they would have to help with the tablet.” (Lily) - “If we enjoy using the technology, we will encourage other staff to use it.” (Vera) - “Staff are having ‘consultation fatigue’. They are consulted on various things. It is sometimes difficult to engage them.” (Cindy)	A study demonstrated the positive changes in nursing staff’s perceptions towards a robotic seal, and staff started incorporating the robot into their routines after observing the therapeutic effects of the robot with the residents in LTC [46].
**T - Tailor planning with staff partners** Tailor various plans on engagement, training, technical support and debriefing schedules	- “I am a visual learner. I need to practice and practice a few more times before I get familiarized with how to do it. Practice is critical besides verbal or written education.” (Sophie) - “If there are technical issues, staff can contact someone. It will increase the feeling of confidence of staff to use it.” (Britney) - “Certain boundaries and guidelines will be needed to prevent invasion of privacy.” (Sophie)	Yuan et al. [39] emphasized the diversity of care staff skills and motivations when introducing social robots to staff in LTC.
**A - Acknowledge staff concerns** Dedicate time to acknowledge and address staff concerns and offer a psychologically safe space	- “Create open spaces to ask about staff concerns and validate them… the closer you are physically to staff, the more engaged and grounded approaches that you use, the more responses that you will get.” (Mandy) - “If we need to assist and give cues to those residents who are not good at interacting in the conversation, it will take us much time and create burdens. We still need to care about other residents.” (Rocky)	Koh et al. [45] demonstrated how joint discussions with colleagues allowed staff to express their concerns and empower each other with skills to manage the negative reactions of residents towards the pet robots.
**R – Recruit opinion leaders early** Recruit opinion leaders and staff champions who support the interventions early in the implementation	- “Engaging those who are interested and see the potential benefits of using the robots… Sometimes you just cannot get the buy-ins from certain staff.” (Johnny) - “Get support from different levels of leadership and leaders representing different groups, for example, leaders of the family council, residents’ council, and operational leaders. They can be helpful to generate engagement.” (Janet)	Getson and Nejat [40] suggested that all stakeholders should be considered in technology implementation. Identifying champions among staff members was an enabling factor for the successful implementation of technology in LTC homes.
**T - Target residents’ needs** Implementation of interventions should target and match residents’ needs. The implementation team needs to evaluate the impact of the intervention and adjust interventions or implementation strategies accordingly.	- “Robots can create private spaces for family and residents for their conversations. They can share intimate information without staff assisting and staying in the room.” (Mandy) - “For a person who speaks a different language, staying connected with somebody who understands them [through the robot] would be really good.” (Amy) - “The robots are now more urgently needed when in-person visits are not allowed.” (Katherine)	The perceived benefits of addressing residents’ needs will become an internal drive and motivation for staff to adopt using the robots in their routines [39].

### Strengths and Limitations

Participants from different disciplines strengthens the exploration of implementation processes in complex healthcare settings like LTC, which usually involve partnerships between disciplines. Using CFIR to guide the research process such as guiding the development of interview questions and data analysis ensured all domains of intervention implementation to be considered. We have included patient partners with dementia in our research process who provided their lived experiences perspectives. However, the participants were limited to two Canadian LTC, limiting the representation of perspectives of the workforce. Due to the specific context, the findings may not be generalizable to LTC in other countries. Data concerning the participants’ racial and age demographics were not captured, limiting the analysis to compare cultural and age differences. Due to COVID-19 pandemic and staff availability, some interviews needed to be conducted virtually, which limited the introduction of telepresence robots to the participants. Interviewees could have a better understanding of how the telepresence robots worked if there was a in-person interaction session with the robots instead of using the introduction video alone.

## Conclusions

Frontline healthcare staff are at the forefront of applying technology-based solutions to drive healthcare transformation, improve health outcomes and enhance patients and caregivers’ experience amid the current pandemic environment and beyond. To enhance the generalisability of QI methods like the PDSA cycle to a broader context, the potential of using IS with QI methods should be explored. We illustrate how CFIR, an important framework in IS, can act as supplementary support to the PDSA cycle and offer a pragmatic resource – ‘START’ for supporting healthcare leaders in implementing innovations in LTC.

## Supplementary Information

**Additional file 1.**

## Data Availability

The datasets used and/or analyzed during the current study are available from the corresponding author upon reasonable request.

## Abbreviations

CFIR: Consolidated Framework for Implementation Research
COI: Characteristics of Individuals
IC: Intervention Characteristics
IS: Inner Setting
LTC: long-term care
OS: Outer Setting
P: Process
PDSA: Plan-Do-Study-Act
SRQR: Standards for Reporting Qualitative Research
