# Supplementary material for: Beyond Plan-Do-Study-Act cycle – staff perceptions on facilitators and barriers to the implementation of telepresence robots in long-term care
Source: BMC Health Serv Res. 2023 Jul 19;23:772. doi: 10.1186/s12913-023-09741-9 (PMC10357815; doi:10.1186/s12913-023-09741-9)
Supplement: Supplementary file 1 — Additional file 1. [file 12913_2023_9741_MOESM1_ESM.docx]

| S1: P. 1 |
| --- |
| S2: P. 2 |
| S3: P.3-6 |
| S4: P.6 L15-20 |
| S5: P.6-8 |
| S6: P.7  L3-4 |
| S7: P.7 L12-14 |
| S8: P.7 L16-22 |
| S9: P.9  L8-11 |
| S10: P.8, P.9 L1-6 |
| S11: P.8, L5-9 |
| S12: P.7-8, P.10, L2-6 |
| S13: P.9, L10-22 |
| S14: P.9-10 |
| S15: P.9 |
| S16: P.10-14, 16-17 |
| S17: P.10-14, 16-17 |
| S18: P.14-18 |
| S19: P.18 |
| S20: P.19 |
| S20: P.19 |

Appendix 1. Checklist for the Standards for Reporting Qualitative Research (SRQR)


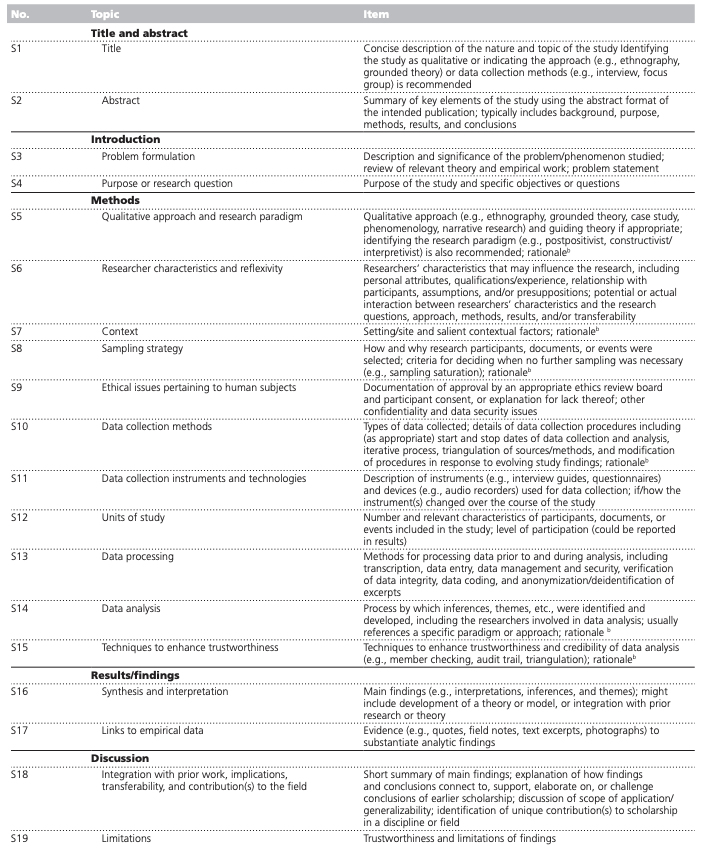

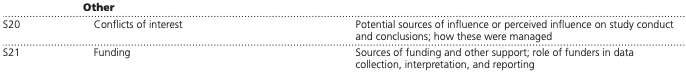


References:

O’Brien BC, Harris IB, Beckman TJ, Reed DA, Cook DA. Standards for reporting qualitative research: A synthesis of recommendations. Academic Medicine [Internet]. 2014 [cited 2022 Jul 18];89(9):1245–51. Available from: https://journals.lww.com/academicmedicine/Fulltext/2014/09000/Standards_for_Reporting_Qualitative_Research__A.21.aspx
